# Supplementary material for: Proteomic screening of TMEM43 binding partners identifies VDAC leading to mitochondrial dysfunction
Source: PLoS One. 2025 Dec 18;20(12):e0339129. doi: 10.1371/journal.pone.0339129 (PMC12714281; doi:10.1371/journal.pone.0339129)
Supplement: S1 File — The original images of all immunobloting. (PDF) [file pone.0339129.s014.pdf]

The original images of immunoblotting

Fig 4C. VDAC2 IP

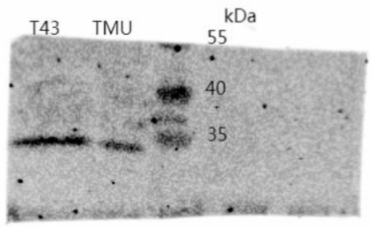

Fig 4C. VDAC1 IP

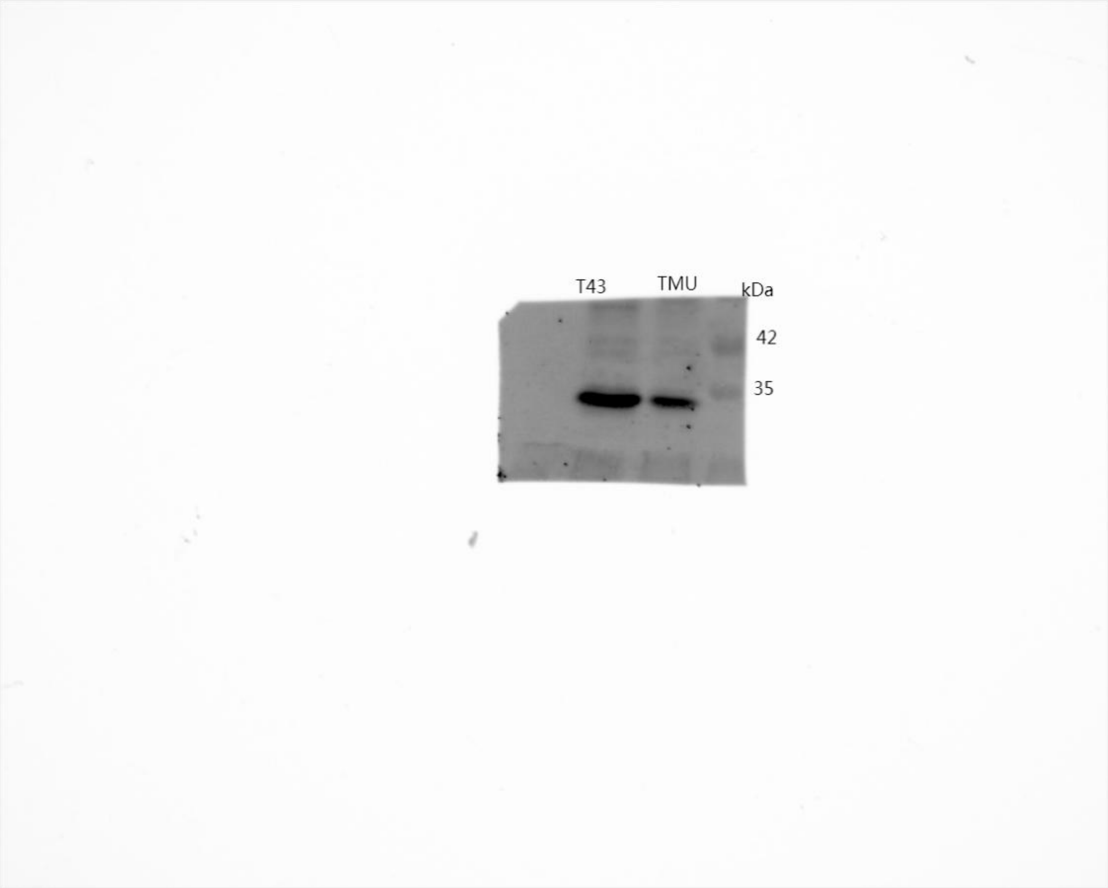

Fig 4C. Flag IP

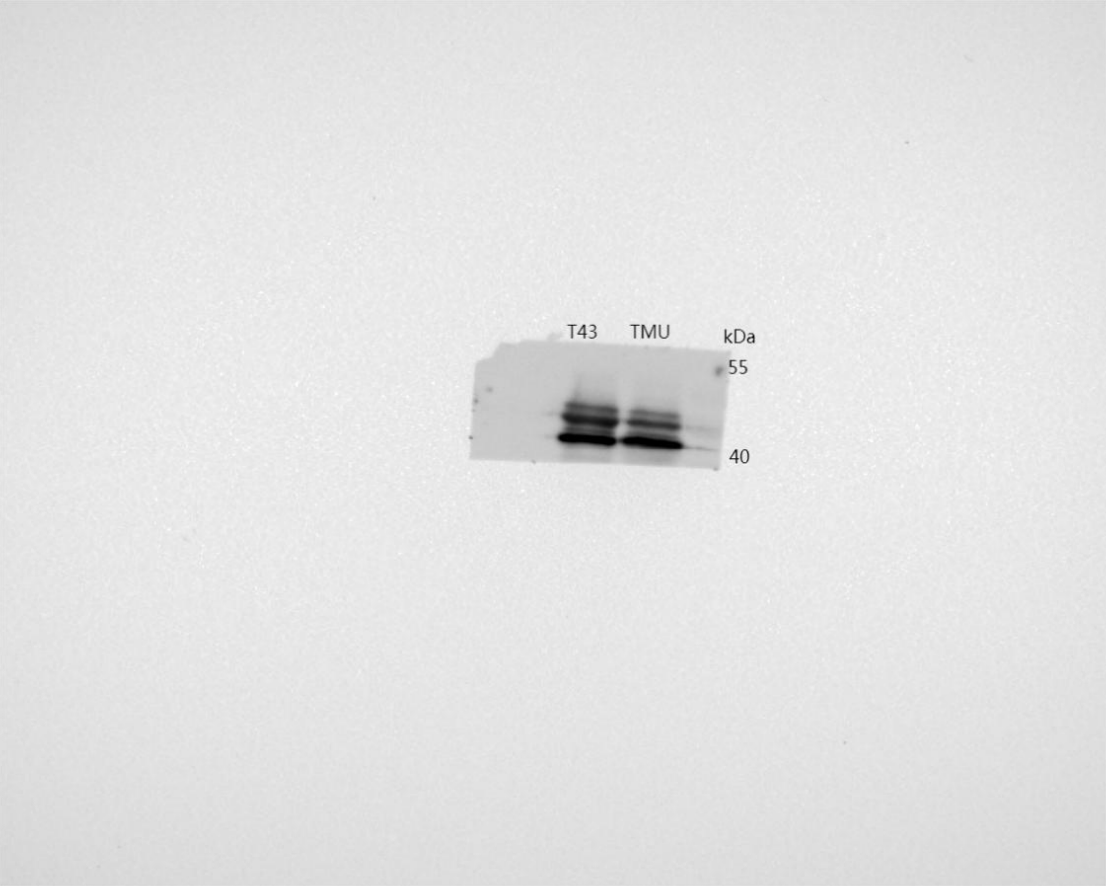

Fig 4C. Flag input

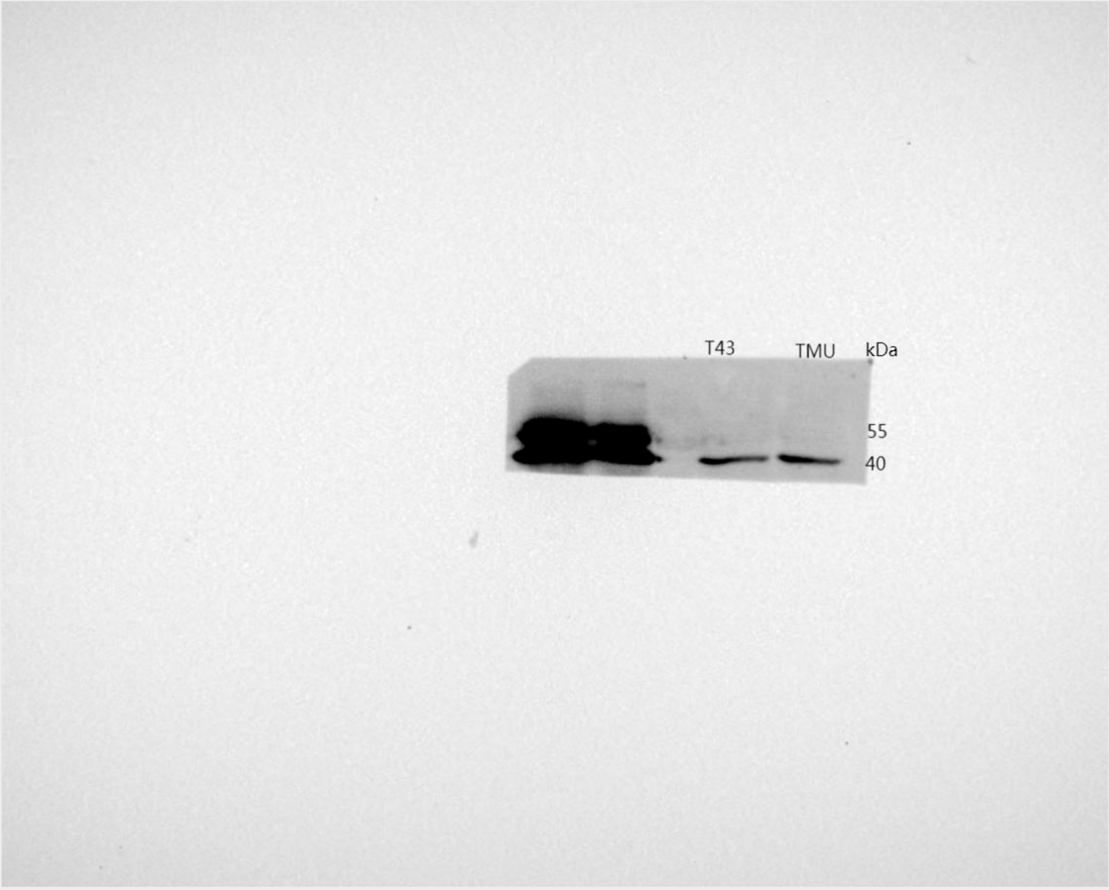

Fig 4C. VDAC1 input

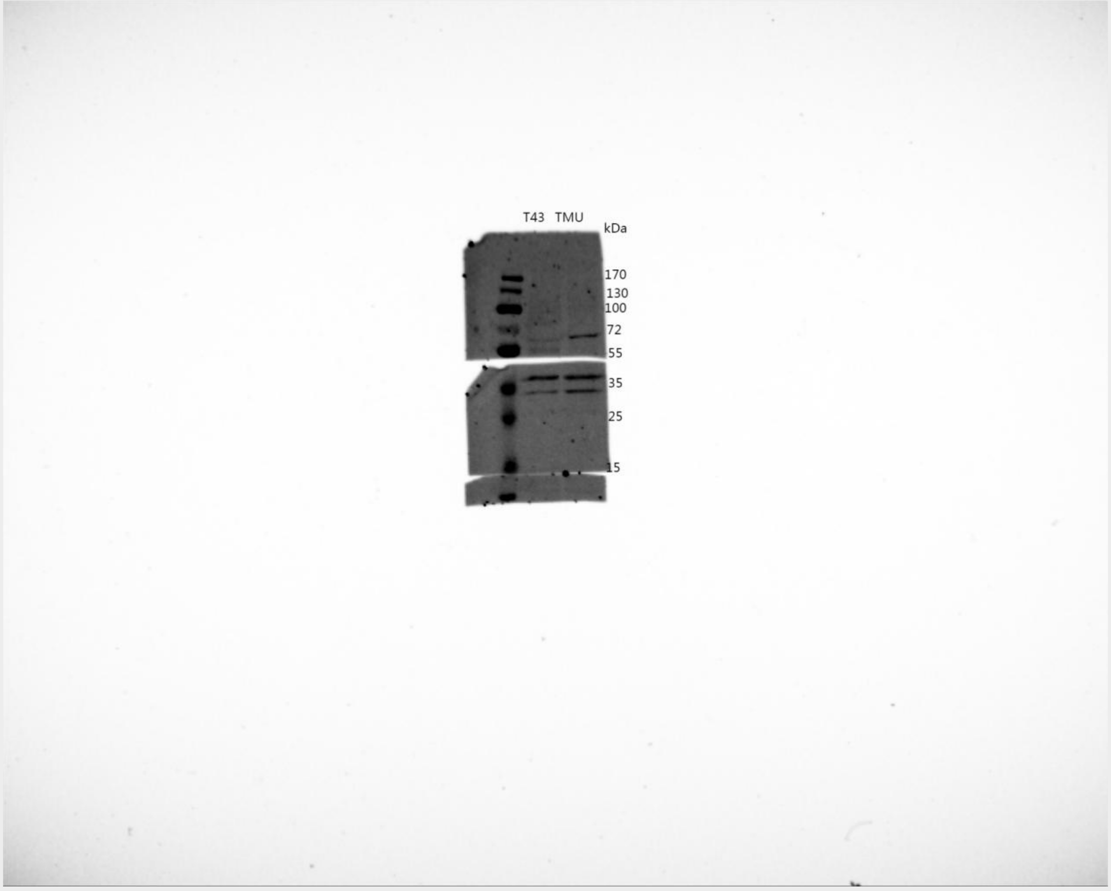

Fig 4C. GAPDH input

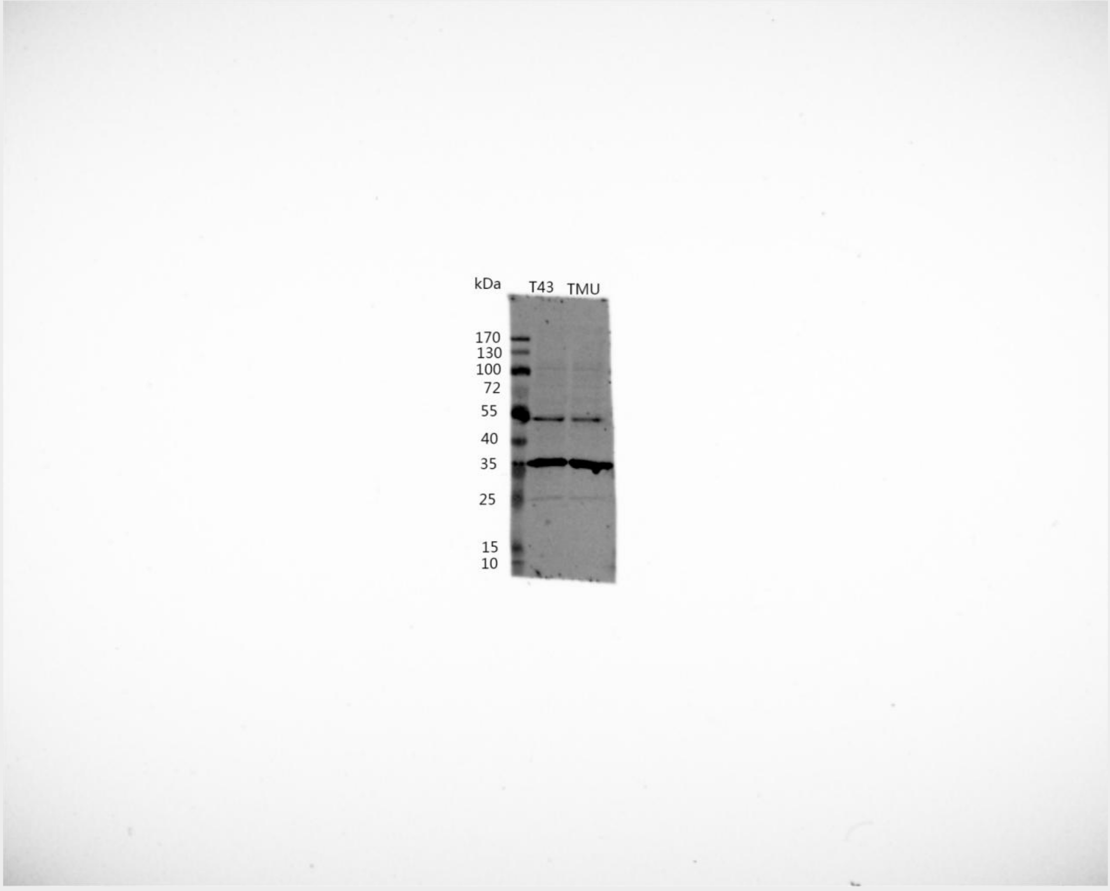

Fig 4C. VDAC2 input

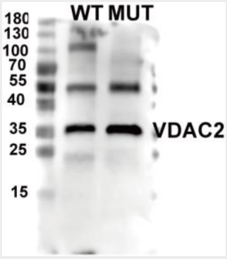

Fig 4C. IgG IP

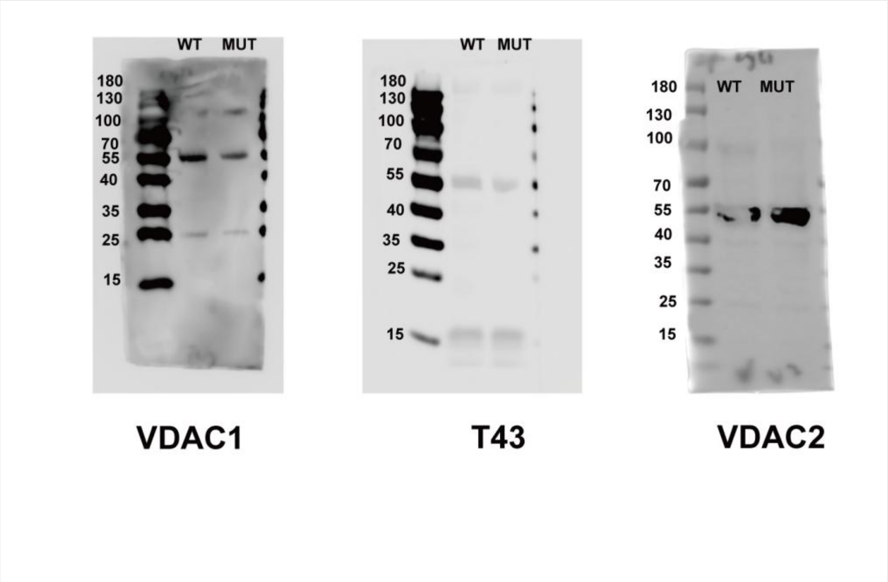

Fig 4D. VDAC1_IP

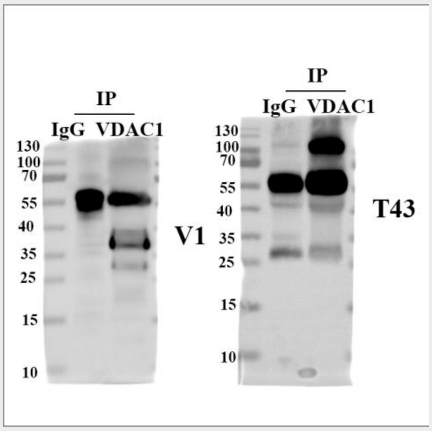

Fig 4D. T43_IP

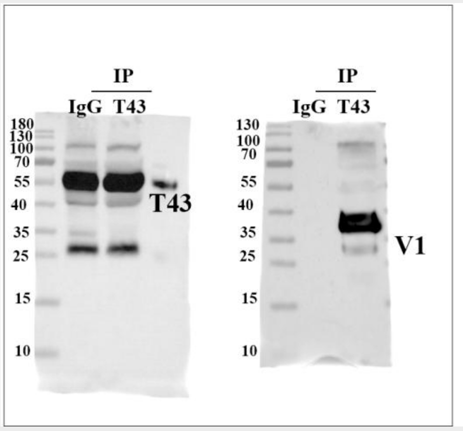

Fig 4D. VDAC1_INPUT

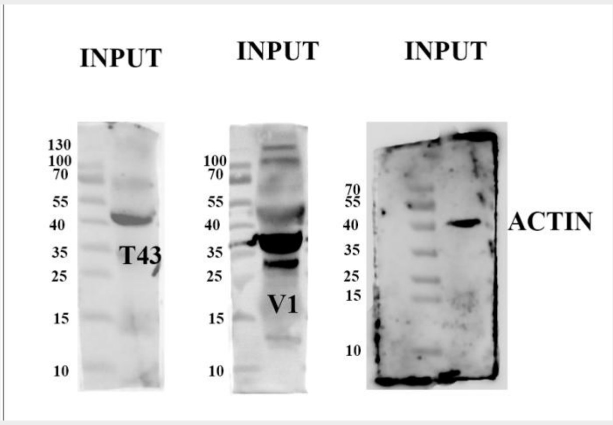

Fig S1A. HA blotting of TMEM43 mutant cell IP

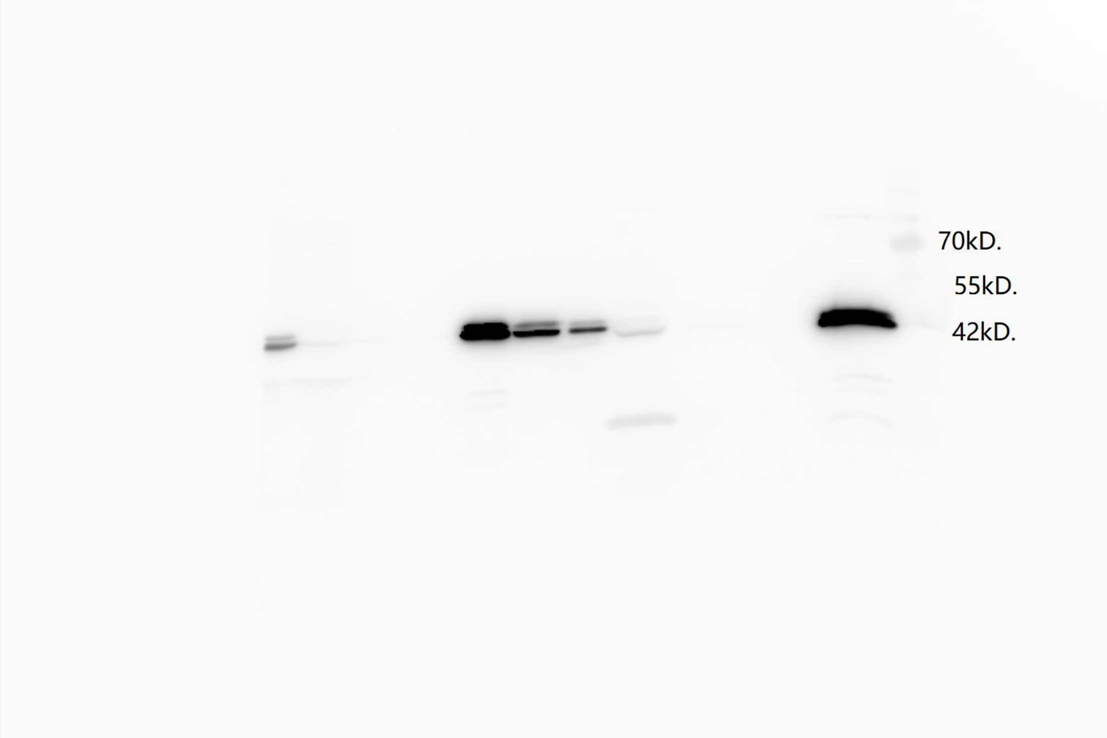

Fig S1A. HA blotting of TMEM43 cell IP

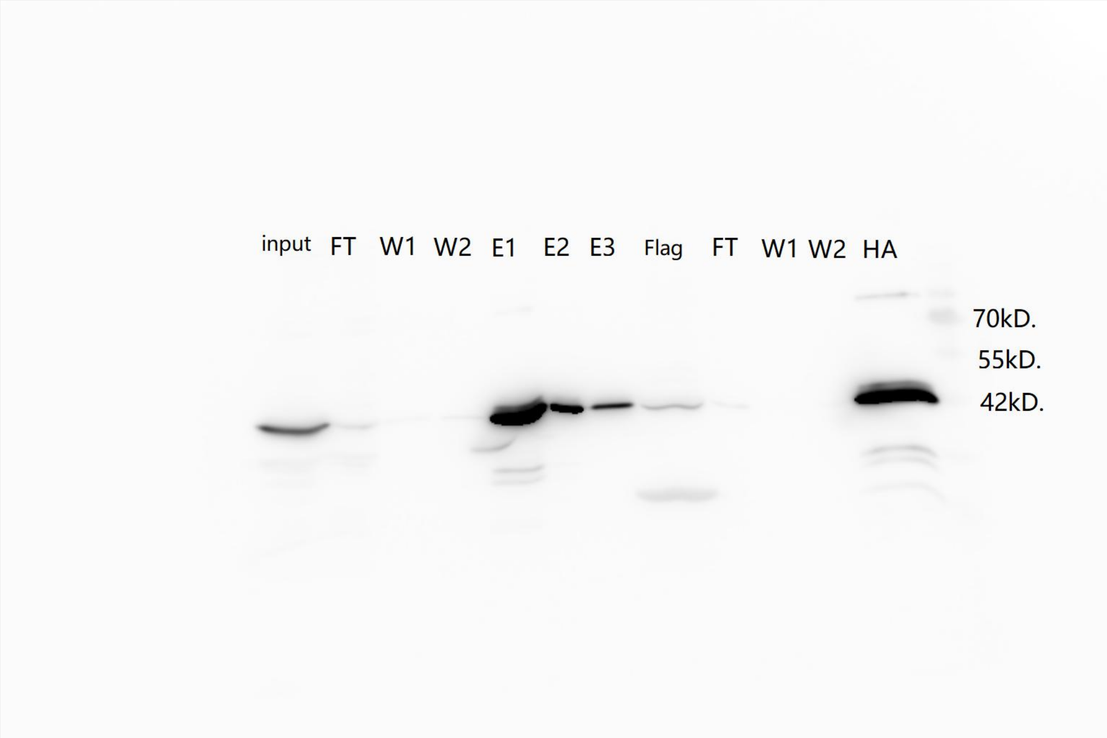

Fig S1A. HA blotting of vector cell IP

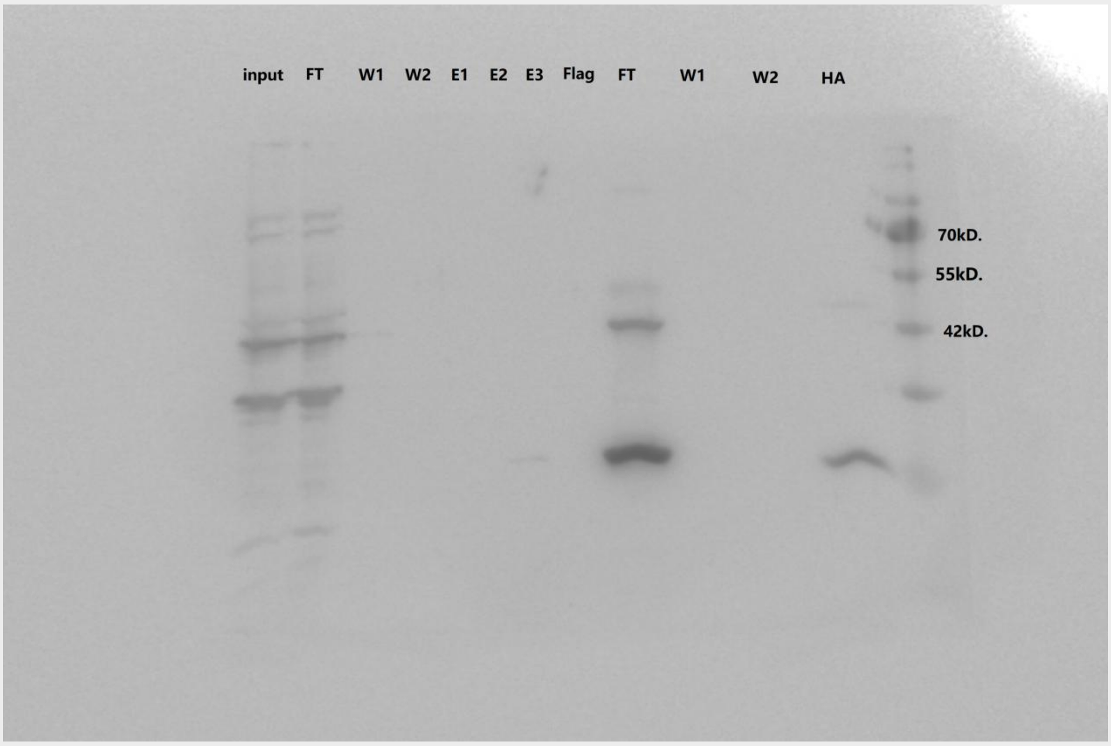

Fig S1B.

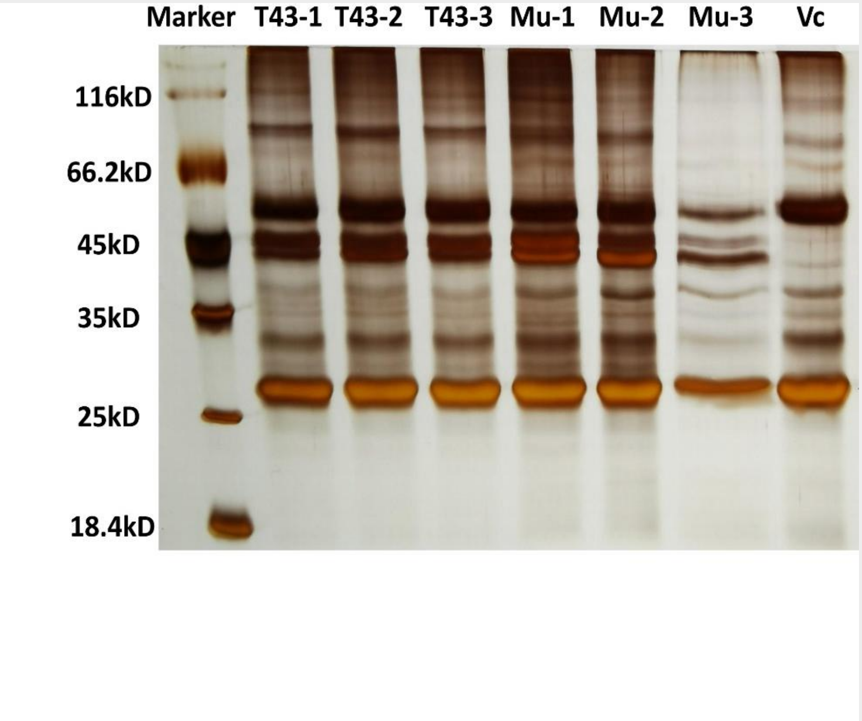
Marker
T43-1
T43-2
T43-3
Mu-1
Mu-2
Mu-3
Vc
116kD
66.2kD
45kD
35kD
25kD
18.4kD

Fig S4A

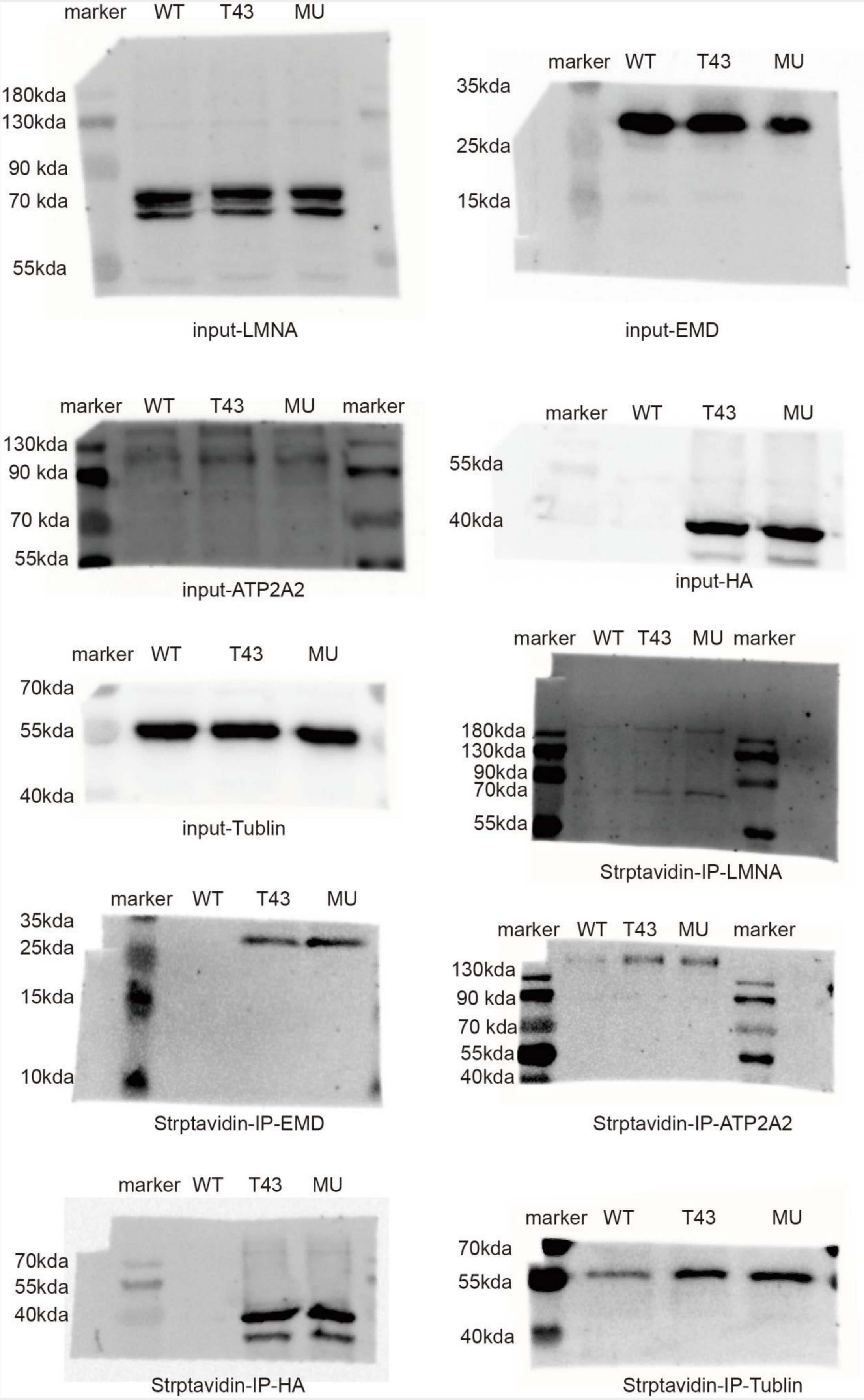
marker WT T43 MU
180kda
130kda
90 kda
70 kda
55kda
input-LMNA
marker WT T43 MU
35kda
25kda
15kda
input-EMD
marker WT T43 MU marker
130kda
90 kda
70 kda
55kda
input-ATP2A2
marker WT T43 MU
55kda
40kda
input-HA
marker WT T43 MU
70kda
55kda
40kda
input-Tublin
marker WT T43 MU marker
180kda
130kda
90kda
70kda
55kda
Strptavidin-IP-LMNA
marker WT T43 MU
35kda
25kda
15kda
10kda
Strptavidin-IP-EMD
marker WT T43 MU marker
130kda
90 kda
70 kda
55kda
40kda
Strptavidin-IP-ATP2A2
marker WT T43 MU
70kda
55kda
40kda
Strptavidin-IP-HA
marker WT T43 MU
70kda
55kda
40kda
Strptavidin-IP-Tublin

Fig S6A. IP_FLAG

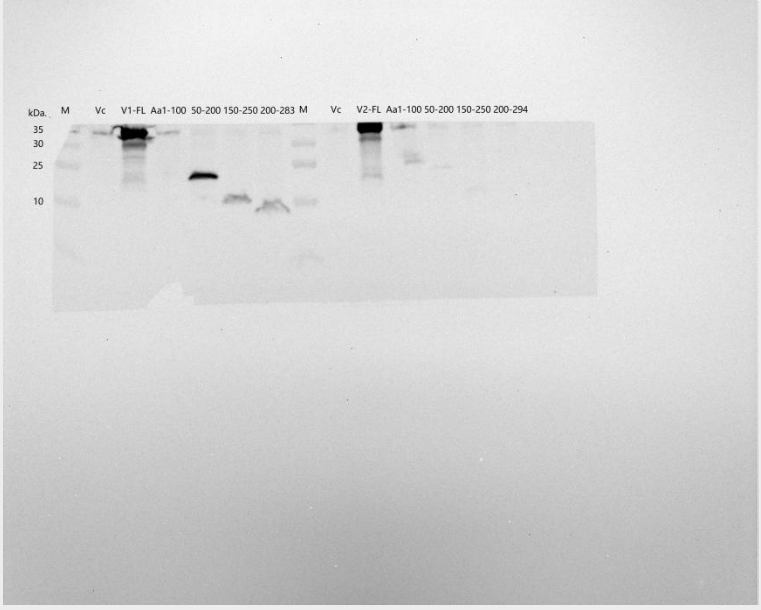

Fig S6A. IP_HA

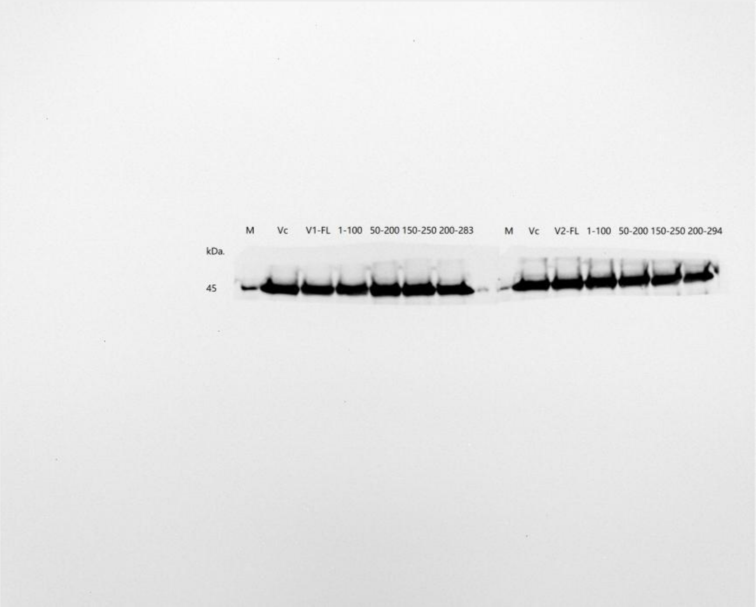

Fig S6A. Input_HA

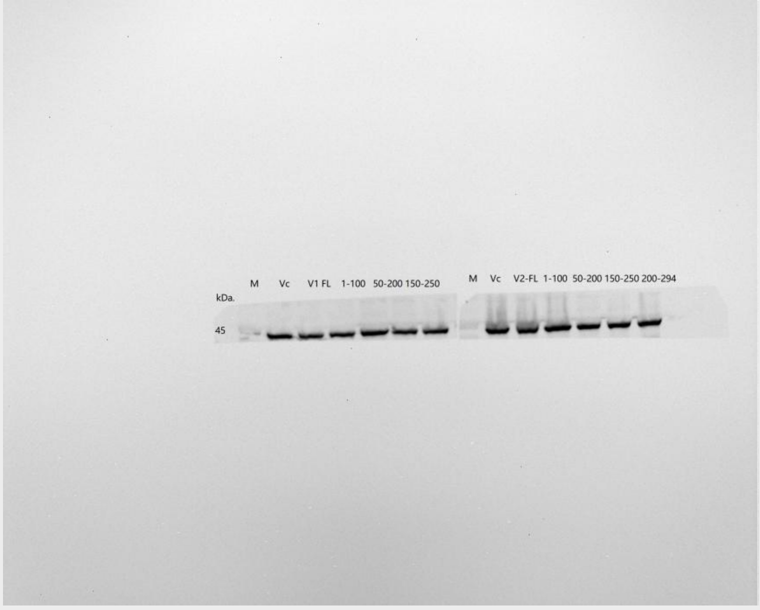

Fig S6A. Input_ACTIN

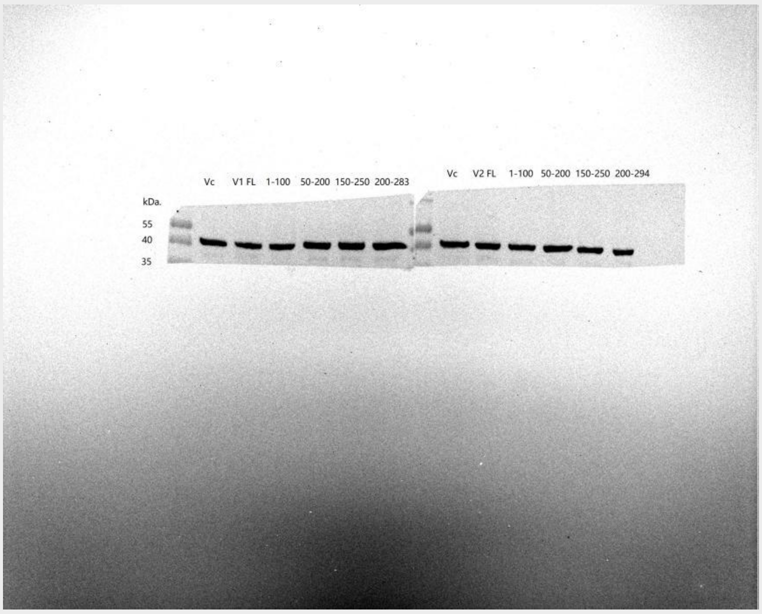

Fig S6B. VDAC1

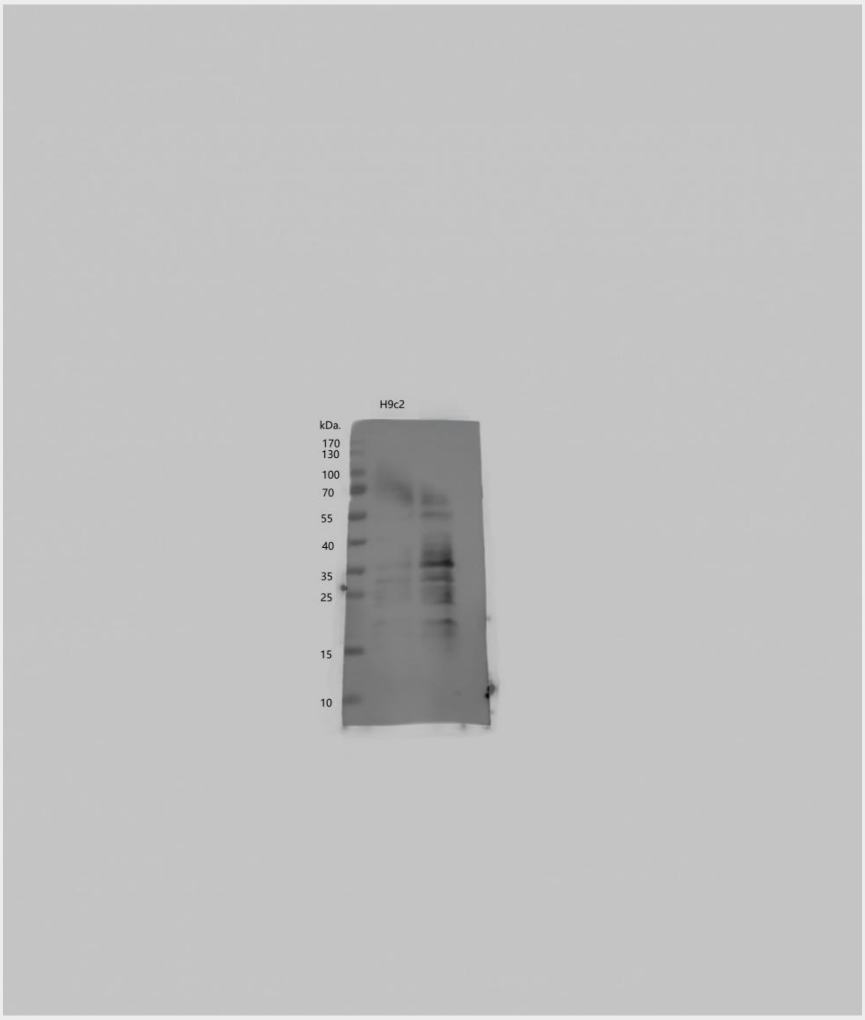

Fig S6B. VDAC2

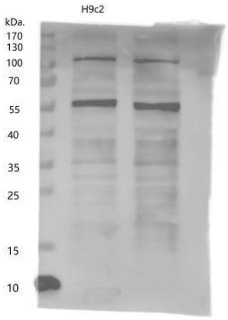

Fig S6B. H3

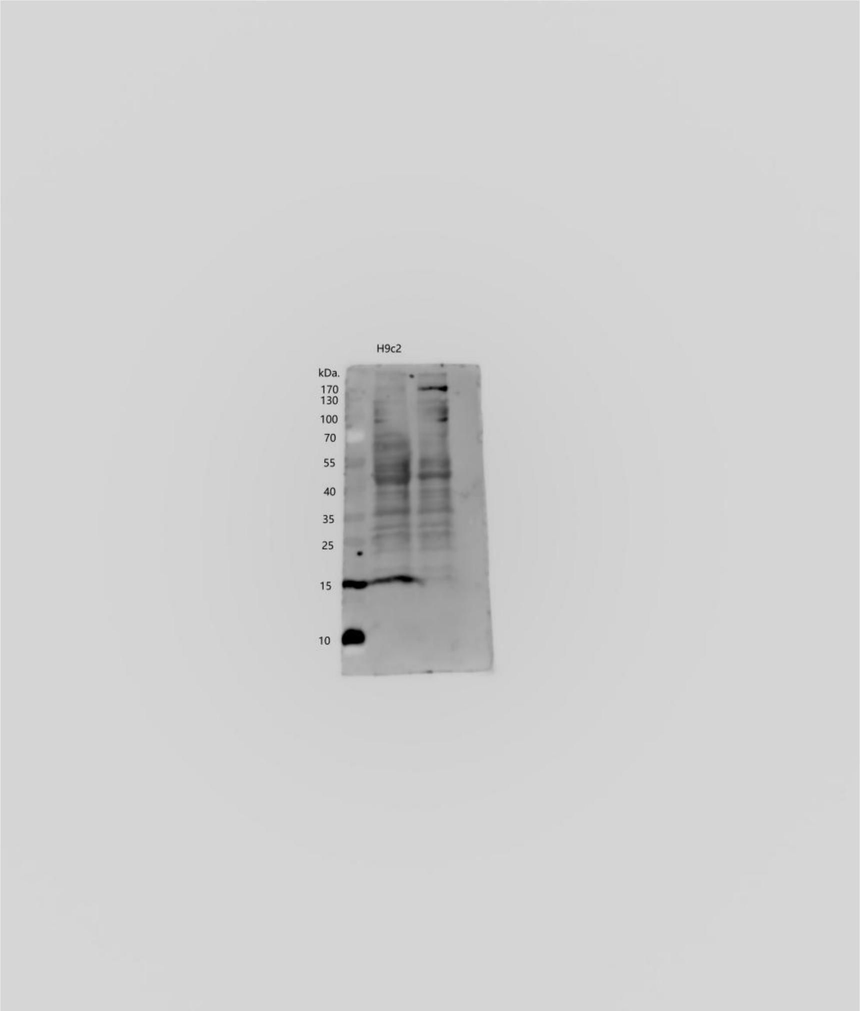

Fig S6C. Actin

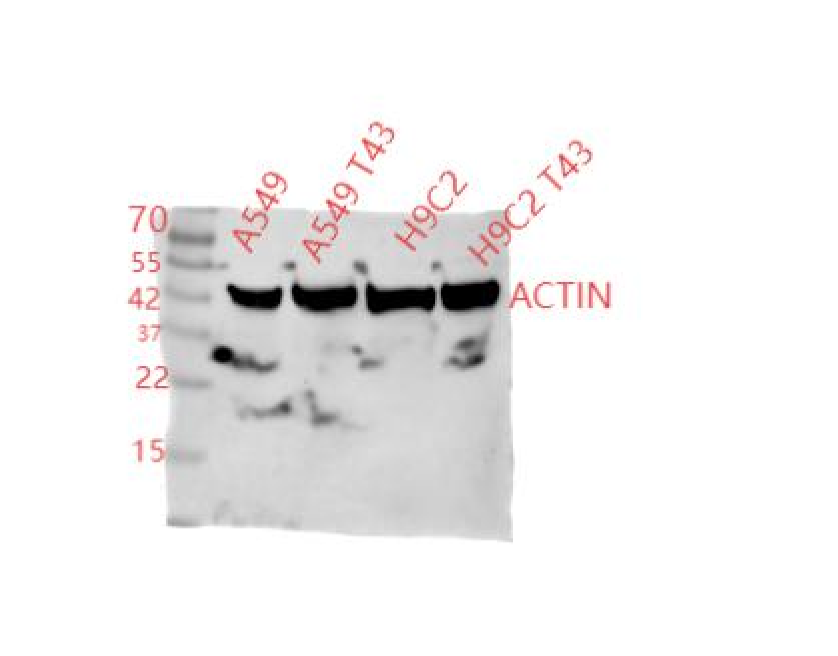

Fig S6C. TMEM43

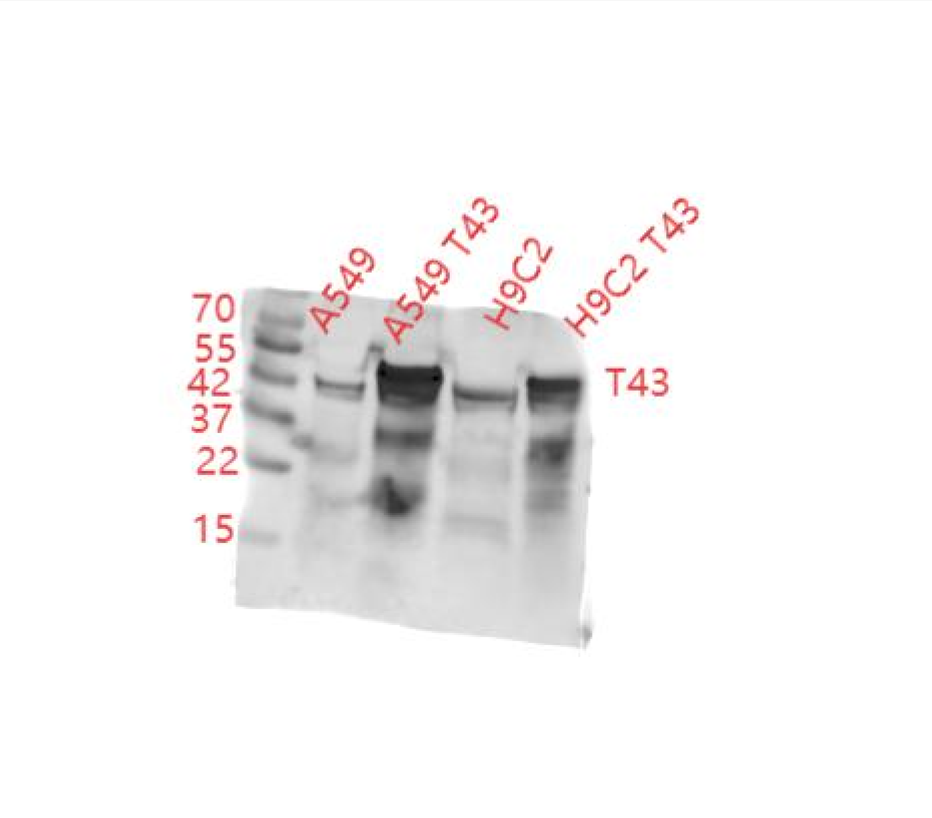

Fig S8

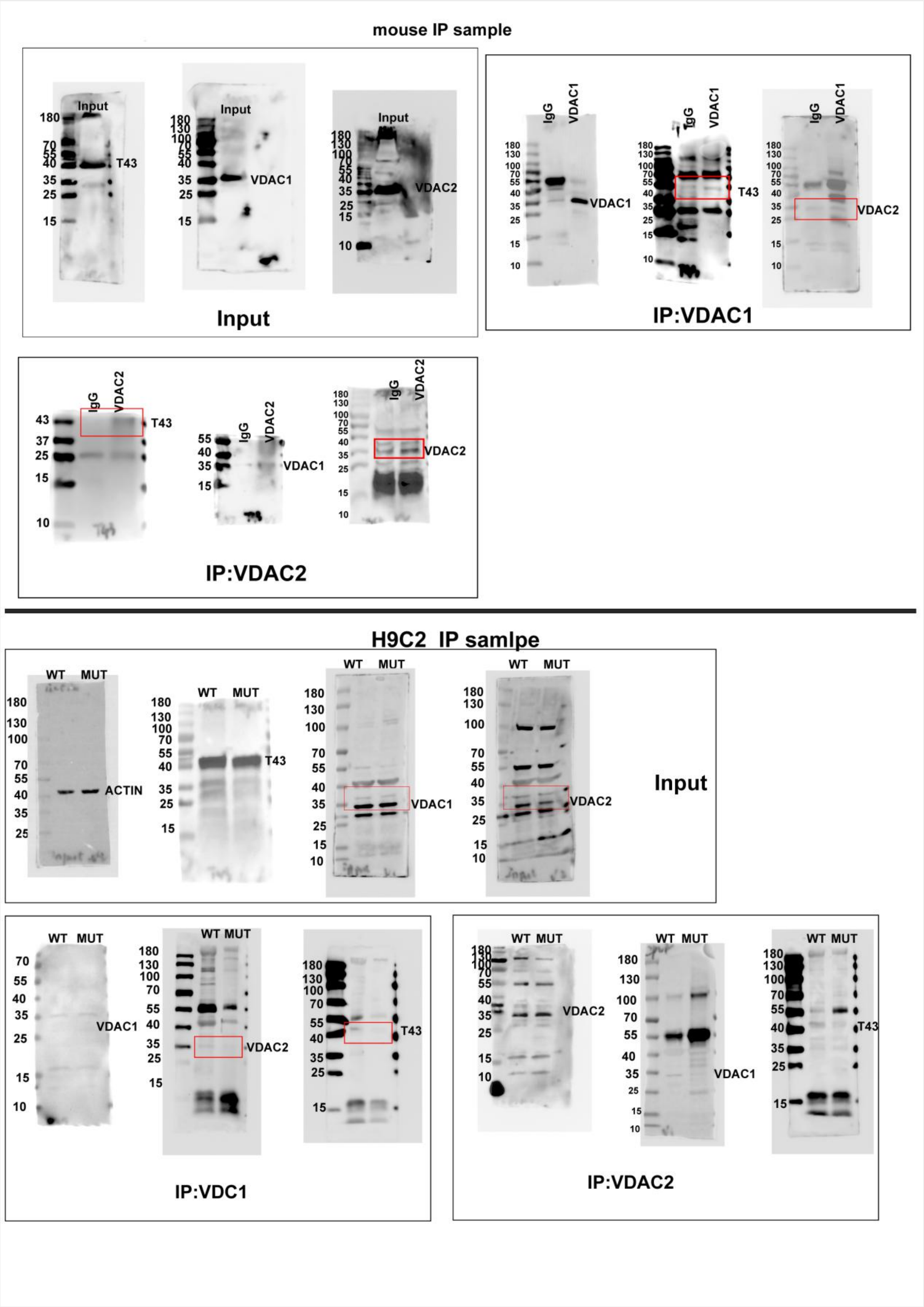

mouse IP sample
Input
T43
VDAC1
VDAC2
Input
IgG
VDAC1
IP:VDAC1
IgG
VDAC2
T43
VDAC1
VDAC2
IP:VDAC2
H9C2 IP samlpe
WT
MUT
ACTIN
T43
VDAC1
VDAC2
Input
WT
MUT
VDAC1
VDAC2
T43
IP:VDC1
WT
MUT
VDAC2
VDAC1
T43
IP:VDAC2

Fig S8C

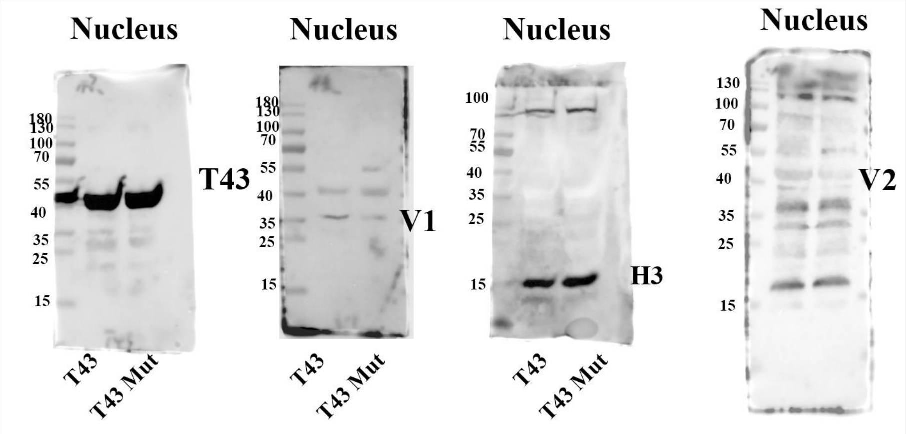
Nucleus
Nucleus
Nucleus
Nucleus
180
130
100
70
55
40
35
25
15
T43
T43
T43 Mut
180
130
100
70
55
40
35
25
15
V1
T43
T43 Mut
100
70
55
40
35
25
15
H3
T43
T43 Mut
130
100
70
55
40
35
25
15
V2
