# Supplementary material for: Proteomic screening of TMEM43 binding partners identifies VDAC leading to mitochondrial dysfunction
Source: PLoS One. 2025 Dec 18;20(12):e0339129. doi: 10.1371/journal.pone.0339129 (PMC12714281; doi:10.1371/journal.pone.0339129)
Supplement: Attachment — Submitted filename: Response to the comments 3.pdf [file pone.0339129.s016.pdf]

Response to the comments

Summary of revisions：

1. Several new experiments were carried out to address the comments including detection of Ca^{2+} level and ROS level in S10 Fig, S8 Fig Reverse IP with VDACs in H9c2 cells and mouse hearts, IgG IP in Fig 4C, WB in S6 Fig et al.
2. Six panels including Fig 3A, Fig 4A, Fig 4C, Fig 4D, Fig 5A, Fig 5B are all updated.
3. Quantity analyses are added in Fig 4B and S5 Fig.
4. New result are provided inS6 Fig, S8 Fig, S9 Fig, S10 Fig and S11 Fig.
5. The marker and sample names are labeled in all original gel images in S1 File.
6. The whole manuscript is revised thoroughly to meet the style requirements of PLOS ONE.

Journal requirements:

When submitting your revision, we need you to address these additional requirements.

1. Please ensure that your manuscript meets PLOS ONE's style requirements, including those for file naming. The PLOS ONE style templates can be found at
https://journals.plos.org/plosone/s/file?id=wjVg/PLOSOne_formatting_sample_main_body.pdf
and
https://journals.plos.org/plosone/s/file?id=ba62/PLOSOne_formatting_sample_title_authors_affiliations.pdf

Response: The manuscript is revised to meet the style requirements of PLOS ONE.

2. Thank you for stating the following financial disclosure:
“This work was supported by grants from Shenzhen Science and Technology Planning Program (JCYJ20210324122814040 to G. Zheng, JCYJ20190809161811237 to J. Zhang and JCYJ20210324104214040 to Yi Lu), National Natural Science Foundation of China (81972420 to Yi Lu and 81972766, 82173336 to J. Zhang) and Chengming Zhu’s start-up funding from Sun Yat-Sen University.”
Please state what role the funders took in the study. If the funders had no role, please state: "The funders had no role in study design, data collection and analysis, decision to publish, or preparation of the manuscript."
If this statement is not correct you must amend it as needed.
Please include this amended Role of Funder statement in your cover letter; we will change the online submission form on your behalf.

Response: The funders had no role, and the statement is added in the manuscript.

3. Your ethics statement should only appear in the Methods section of your manuscript. If your ethics statement is written in any section besides the Methods, please delete it from any other section.

Response: The ethics statement is moved to the Methods section.

4. PLOS ONE now requires that authors provide the original uncropped and unadjusted images underlying all blot or gel results reported in a submission ’ s figures or Supporting Information files. This policy and the journal ’ s other requirements for blot/gel reporting and figure preparation are described in detail at https://journals.plos.org/plosone/s/figures#loc-blot-and-gel-reporting-requirements and https://journals.plos.org/plosone/s/figures#loc-preparing-figures-from-image-files. When you submit your revised manuscript, please ensure that your figures adhere fully to these guidelines and provide the original underlying images for all blot or gel data reported in your submission. See the following link for instructions on providing the original image data: https://journals.plos.org/plosone/s/figures#loc-original-images-for-blots-and-gels.
In your cover letter, please note whether your blot/gel image data are in Supporting Information or posted at a public data repository, provide the repository URL if relevant, and provide specific details as to which raw blot/gel images, if any, are not available. Email us at plosone@plos.org if you have any questions

Response: The original uncropped and unadjusted images are provided in the Supporting Information S1 File, please check it.

Additional Editor Comments:

While the authors have provided clear and detailed methodologies, there are major concerns raised by the Reviewers that must be addressed. I concur that additional, extensive, and repeated experiments are necessary to strengthen the validity of the findings. To substantiate their claims, the authors must not only address the Reviewers' comments but also provide conclusive evidence that the TMEM43 S358L mutation causes ARVC5. This requires in vivo experiments rather than relying solely on in vitro and ex vivo approaches.

In detail, the authors must provide direct evidence linking the TMEM43 S358L mutation to ARVC. This could be achieved through in vivo studies using a transgenic mouse model that demonstrates this mutation induces the structural, functional, and electrophysiological remodeling characteristic of ARVC5. At the very least, in vitro electrophysiological characterization (e.g., patch-clamp techniques) should be performed to support their claims. At this stage, the assertion that 'characterizing the interacting proteins of TMEM43 mutants is important for unraveling the underlying mechanisms of ARVC5' remains unsubstantiated. While the study explores the relationship between the S358L mutation and VDAC1/VDAC2, this connection alone does not establish causality or provide sufficient insight into whether this mutation is indeed responsible for ARVC5.

Response: The direct evidence in rodent models linking the TMEM43 S358L mutation to ARVC has been published by 3 different groups (Protein&Cell 2019 knock-in mice [1]; Circulation 2019 transgenic mice [2]; American Journal of Physiology Heart and Circulatory Physiology 2023 knock-in mice [3]). The structural abnormalities of TMEM43 S358L mice in all 3 publications

contained significantly higher ratio of heart weight to body weight (HW/BW), progressive cardiac dilatation, and faster fibrofatty progression. The echocardiography analyses of cardiac functions showed reduced left ventricular ejection fraction (LVEF) in mutant mice. The electrocardiograms of mutant mice [2, 3] exhibited prolonged QRS interval, and other abnormalities like decreasing QRS amplitude, premature atrial and ventricular contractions, and intolerance strenuous running stress. Moreover, human induced pluripotent stem cell (hiPSC) derived cardiomyocytes bearing the p.S358L mutation [2] showed increased contraction duration, decreased contraction amplitude, and slower Ca2+ transient upon isoproterenol treatments. It is interesting that TMEM43 interacted with VDAC1 in other cell lines [4]. Furthermore, the mitochondria abnormalities were discovered in the mouse hearts carrying TMEM43 S358L mutation [3]. Whatever, the assertion is revised.

Reference：

[1] Zheng G, Jiang C, Li Y, Yang D, Ma Y, Zhang B, Li X, Zhang P, Hu X, Zhao X, Du J, Lin X. TMEM43-S358L mutation enhances NF-κB-TGFβ signal cascade in arrhythmogenic right ventricular dysplasia/cardiomyopathy. Protein Cell. 2019 Feb;10(2):104-119. doi: 10.1007/s13238-018-0563-2. Epub 2018 Jul 6. PMID: 29980933; PMCID: PMC6340891.

[2] Padrón-Barthe L, Villalba-Orero M, Gómez-Salinero JM, Domínguez F, Román M, Larrasa-Alonso J, Ortiz-Sánchez P, Martínez F, López-Olañeta M, Bonzón-Kulichenko E, Vázquez J, Martí-Gómez C, Santiago DJ, Prados B, Giovinazzo G, Gómez-Gaviro MV, Priori S, Garcia-Pavia P, Lara-Pezzi E. Severe Cardiac Dysfunction and Death Caused by Arrhythmogenic Right Ventricular Cardiomyopathy Type 5 Are Improved by Inhibition of Glycogen Synthase Kinase-3β. Circulation. 2019 Oct;140(14):1188-1204. doi: 10.1161/CIRCULATIONAHA.119.040366. Epub 2019 Sep 5. PMID: 31567019; PMCID: PMC6784777.

[3] Orgil BO, Munkhsaikhan U, Pierre JF, Li N, Xu F, Alberson NR, Johnson JN, Wetzel GT, Boukens BJD, Lu L, Towbin JA, Purevjav E. The TMEM43 S358L mutation affects cardiac, small intestine, and metabolic homeostasis in a knock-in mouse model. Am J Physiol Heart Circ Physiol. 2023 Jun 1;324(6):H866-H880. doi: 10.1152/ajpheart.00712.2022. Epub 2023 Apr 21. PMID: 37083466; PMCID: PMC10190833.

[4] Zhang N, Wang F, Yang X, Wang Q, Chang R, Zhu L, Feitelson MA, Chen Z. TMEM43 promotes the development of hepatocellular carcinoma by activating VDAC1 through USP7 deubiquitination. Transl Gastroenterol Hepatol. 2024 Jan 25;9:9. doi: 10.21037/tgh-23-108. PMID: 38317750; PMCID: PMC10838614.

5. Review Comments to the Author

Please use the space provided to explain your answers to the questions above. You may also include additional comments for the author, including concerns about dual publication, research ethics, or publication ethics. (Please upload your review as an attachment if it exceeds 20,000 characters)

Reviewer #1: The authors performed IP-MS between TMEM43 and TMEM43 S358L transfected cells and found a different protein binding profile with TMEM43. Among all the interacted proteins, they identified proteins related to calcium signaling, lipid metabolism, and

cardiovascular diseases. Following in vivo verification, they discovered that VDAC may play a key role in pathogenesis. The mutant TMEM43 group exhibited a decreased binding interaction between VDAC and TMEM. The paper is concise, but significant revisions need to be addressed for further review.

Please see below for details.

Major:

For IP-MS, IgG control is needed for immunoprecipitation (IP) mass spectrometry to help identify false-positive interactions.

Response: Although IgG control is not included, the empty vector controls are contained in IP-MS assay (S1 Table, S2 Table, Fig 1B, S1 Fig). This vector controls help to exclude the false-positive interactions.

A few quantifications are lacking (Fig S4B, S5, 4A). It is recommended that every co-localization IF image should followed by quantification analysis. It is important to point out that the analysis is done in individually successfully plasmid-transfected cells. Due to the existence of endogenous TMEM43, it is difficult to separate the phenotype derived from TMEM43 mutant or endogenously. Making an endogenous model on TMEM43 mutant is highly recommended.

DAPI is often not mentioned in the figure legend where IF was described.

Response: The co-localization IF images are all followed by quantification analyses. DAPI is mentioned in all figure legends of immunofluorescence. We compared the endogenous TMEM43 protein level with overexpression ones and found that the endogenous expression is much lower than the overexpression (S6C Fig). Thus, the effect of endogenous TMEM43 is quite limited due to the very low expression.

Figure 4:

1. VDAC is a transmembrane calcium channel protein expressed in the mitochondrial outer membrane. In Figure 4A, the red fluorescence of VDAC2 is from the nucleus. VDAC1 looks normal.

Response: We checked the cellular location of VDAC2 in Gencards (https://www.genecards.org/) and found that VDAC2 also locates in the nucleus as shown in the following figure.

Subcellular locations from UniProtKB/Swiss-Prot for VDAC2 Gene

Protein: ***VDAC2_HUMAN***

- Mitochondrion outer membrane {ECO:0000269 PubMed:31015432, 7539795}
- Membrane {ECO:0000269 PubMed:27641616}
- Note: May localize to non-mitochondrial membranes {ECO:0000269 PubMed:27641616}

Subcellular locations from COMPARTMENTS

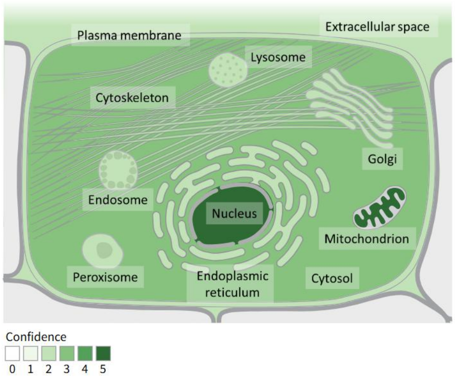

Compartment	Confidence
nucleus	5
mitochondrion	5
cytosol	3
golgi apparatus	2
lysosome	2
endosome	2
endoplasmic reticulum	2
peroxisome	2
cytoskeleton	2
extracellular	2
plasma membrane	2

2. Why is HA localization very different from Figure 3? Isn’t TMEM localized in the nuclear membrane? In Figure 4A, HA is also expressed in the cytosol.

Therefore, the authors can’t make convincing results from Figure 4A.

Response: Although it is not so obvious, HA is also expressed in cytosol in Figure3. The different staining intensity of HA may be due to the difference of cell lines of A549 and H9c2. As indicated by the cellular location of TMEM43 in Gencards (https://www.genecards.org/), the cytosol staining of HA may come from endoplasmic reticulum that surround the nucleus.

Protein: TMM43_HUMAN

- Endoplasmic reticulum membrane {ECO:0000269 PubMed:32614325}
- Nucleus inner membrane; Multi-pass membrane protein
- Cell membrane {ECO:0000269 PubMed:34050020}
- Note: Retained in the inner nuclear membrane through interaction with EMD and A- and B-lamins. hydrophilic domain resides in the endoplasmic reticulum lumen (By similarity) {ECO:0000250}

Subcellular locations from COMPARTMENTS

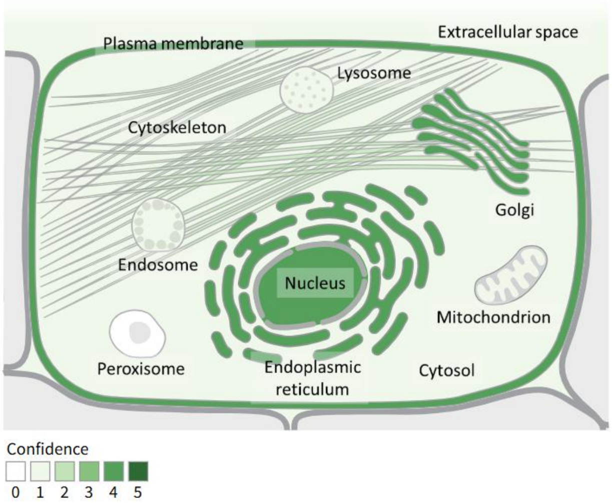

Compartment	Confidence
golgi apparatus	4
endoplasmic reticulum	4
nucleus	4
plasma membrane	4
cytoskeleton	2
lysosome	1
cytosol	1
endosome	1
mitochondrion	1
extracellular	1
peroxisome	0

Figure 4B, IgG control is needed.

Response: The IgG control is added in Fig 4C (original 4B).

Figure 4C, the description is not clear. Labeling what the samples are and what antibodies are used to precipitate is recommended.

For input samples, the TMEM43 density is too low to tell. Either increase the loading amount or use a more sensitive ECL.

Response: Antibodies used to precipitate are labeled. The input of TMEM43 is updated in this figure.

All Western images need to be labeled the molecular size ladder; please refer to Western image publication standards. In the supplementary, please upload all raw images with appropriate labeling.

Response: All original Western images labeled with the molecular size ladder are provided in the supplementary material named S1 File.

To conclude, the decreased interaction between VDAC and TMEM needs to be quantified. Another method is highly recommended to make the conclusion convincing. If authors can make IF results convincing, it also counts. Stain cells with mitochondria maker to see whether mitochondria co-localization with the nucleus decrease may be helpful as VDAC is expressed in mitochondria.

Response: The reverse IP with VDACs in H9c2 and mouse hearts were performed as shown in S8 Fig. Since VDAC is highly expressed in both nucleus and mitochondria, detection of mitochondria co-localization with the nucleus may not help.

Subcellular locations from UniProtKB/Swiss-Prot for VDAC2 Gene

Protein:* *VDAC2_HUMAN

- Mitochondrion outer membrane {ECO:0000269 PubMed:31015432, 7539795}
- Membrane {ECO:0000269 PubMed:27641616}
- Note: May localize to non-mitochondrial membranes {ECO:0000269 PubMed:27641616}

Subcellular locations from COMPARTMENTS

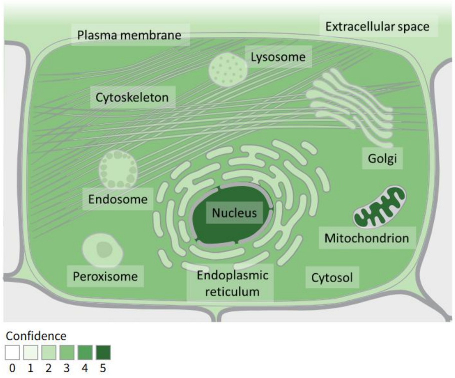

Compartment	Confidence
nucleus	5
mitochondrion	5
cytosol	3
golgi apparatus	2
lysosome	2
endosome	2
endoplasmic reticulum	2
peroxisome	2
cytoskeleton	2
extracellular	2
plasma membrane	2

Figure 4A, I recommend having an individual channel in addition to a merged one; for example, the red fluorescence in the left bottom is too dim to tell whether there is some localization on the nuclear membrane or TMEM43.

Response: The individual channel of Figure 4A are provided in S9 Fig in the file of supplementary materials.

There are limitations of IF and IP. IF is a 2D image, and proteins are inseparable between the cytosol and organelles if a generic protein lysis method is used. To make the data more convincing, nuclear protein-specific isolation is recommended to test whether the mutant group's intersection between VDAC and TMEM43 is decreased.

Response: We tried to isolate the nucleus in H9c2 cells and detected the protein level of VDACs. However, the signal is quite weak through Western blot (S6B Fig). Thus, there is technical problem to compare the nuclear protein level of VDACs in T43 and Mu cells.

Figure 5:

A did not label what violet signal is.

Line 467, it is abrupt to mention TMEM43 function in mitochondria, it is not clearly described in the context.

Response: Blue is DAPI staining, which is mentioned in figure legend. The description in the context is modified.

B and C

Analysis should be done in the cells that have the plasmid transfected. Not all cells have the TMEM mutant plasmid.

Response: We are confused, please make it clear.

Figure B needs a higher resolution to indicate that JC-1 is in the mitochondria to prove its efficacy.

Response: Figure 5B is updated.

Minor:

It is vague to say:

"IF staining of TurboID H9c2 cells revealed that biotin and HA labeled TMEM43 were completely overlapping (Fig. S5), indicating the precision of TurboID system."

Are authors implying Turbo ID system consistency with Flag-tagged TMEM43 used before, since plasmids in both systems are HA-tagged?

The expression plasmid information in the flag tagged TMEM43 has not been revealed.

Response: It means that TurboID system is consistent to IF staining. The description is revised. The plasmid information of Flag tagged TMEM43 is provided in the section of material and methods, that is the lentivirus p2k7 vector carrying HA-Flag-TMEM43 or HA-Flag-TMEM43 S358L mutant.

Reviewer #2: The authors use IP-MS technique and identify 166 interacting protein candidates of TMEM43 mutants. They further confirm that TMEM43 mutant reduce its interaction with VDAC, which mediates mitochondrial dysfunction in cardiac myoblast H9c2 cells of TMEM43 pS358L. This work unravels the underlying mechanisms of TMEM43 mutant in ARVC5, and implies a critical role of mitochondria in ARVC pathology.

However, there are several minor concerns should be considered:

-VDAC is a mitochondrial outer membrane protein, the authors should explain the possibility how VDAC interacts with TMEM43.

Response: We checked the cellular locations of VDAC in Gencards (https://www.genecards.org/) and found that the expression of VDACs are highest both in the mitochondria and nucleus with confidence 5. This indicates that VDAC may interacts with TMEM43 in the nucleus.

-In Figure 4B, the input of VDAC2 is missing.

Response: VDAC2 input is added.

-In Figure 4C, the input of VDAC1 in TMEM43 group and all the TMEM43 bands are weak. The authors should explain this or repeat this experiment.

Response: The figure is updated.

-The IP of TMEM43 with VDAC2 should also be added.

Response: The result is shown in S8 Fig. The endogenous IP of VDACs in H9c2 and mouse hearts work not very well.

-The mitochondrial matrix Ca^{2+} level should be detected.

Response: The mitochondrial matrix Ca^{2+} level was detected through Fluo-4 AM fluorescent probe. The results are shown in S10 Fig.

-In Discussion, "TMEM43 mutant led to 515 mitochondrial fragmentation and mitochondrial membrane potential loss". The role of mitochondrial permeability transition pore should be discussed.
Response: The role of mitochondrial permeability transition pore is added in Discussion at line 524-526.

Reviewer #3: The Present manuscript authors found that mutant TMEM43 shows reduced interaction with the VDAC which eventually causes the mitochondria dysfunction. The work was conducted thoroughly, and the manuscript was crafted well. However, there are some key concerns before considering it for publication.

1. The manuscript title states, "Proteomic screening of TMEM43 binding partners identifies VDAC leading to mitochondrial dysfunction in Arrhythmic Right Ventricular Cardiomyopathy." However, the study primarily focuses on the interaction differences between WT and mutant TMEM43, which is essentially a protein characterization study. Since the research was not conducted using an ARVC mouse model or patient primary cells, I suggest that the authors revise the title accordingly.
Response: ARVC is removed from the title.

2. Please correct the mistake of cell number in line number 250.
Response: Thank the reminding, it is corrected at line236 in updated manuscript.

3. Figure 3: It is recommended to include a control image (immunofluorescence performed in cells without the HA tag) and provide high-quality images to enhance the overall quality of the manuscript.
Response: The IF staining of Vector (Vc) control is provided in Fig 3A.

4. Figure 4A: The VDAC1&2 immunofluorescence shows mitochondrial puncta formation, indicating that the cells were not in a healthy state. I recommend that the authors include images where VDAC1&2 immunofluorescence demonstrates the mitochondrial network.
Response: Fig 4A is updated. The mitochondrial network of VDAC1&2 immunofluorescence are shown in S11 Fig.

5. Figures 4B & 4C: I strongly recommend that the authors perform a reverse IP using VDAC1&2 in H9c2 cells and mouse heart lysate.
Response: The reverse IP using VDAC1&2 antibody in H9c2 cells and mouse hearts were performed as shown in S8 Fig.

6. Figure 5A: It would be beneficial to include high-quality images where mitochondrial morphology is clearly visible, with a greater number of cells.
Response: This figure is updated.

7. Figure 5: It is recommended to measure ROS levels in both the mutant and wild-type, as it is a key hallmark of mitochondrial dysfunction.

Response: The mitochondrial ROS level was detected through MitoSOX treatment. The results are shown in S10 Fig.

8. In line number 471, JC-1 staining is incorrectly referred to as immunofluorescence. Please correct the sentence accordingly.

Response: It has been corrected in the manuscript at line 478 in updated manuscript.
