# Supplementary material for: Proteomic screening of TMEM43 binding partners identifies VDAC leading to mitochondrial dysfunction
Source: PLoS One. 2025 Dec 18;20(12):e0339129. doi: 10.1371/journal.pone.0339129 (PMC12714281; doi:10.1371/journal.pone.0339129)
Supplement: Attachment — Submitted filename: plos one review.docx [file pone.0339129.s015.docx]

The authors performed IP-MS between TMEM43 and TMEM43 S358L transfected cells and found a different protein binding profile with TMEM43. Among all the interacted proteins, they identified proteins related to calcium signaling, lipid metabolism, and cardiovascular diseases. Following in vivo verification, they discovered that VDAC may play a key role in pathogenesis. The mutant TMEM43 group exhibited a decreased binding interaction between VDAC and TMEM. The paper is concise, but significant revisions need to be addressed for further review.

Please see below for details.

Major:

For IP-MS, IgG control is needed for immunoprecipitation (IP) mass spectrometry to help identify false-positive interactions.

A few quantifications are lacking (Fig S4B, S5, 4A). It is recommended that every co-localization IF image should followed by quantification analysis. It is important to point out that the analysis is done in individually successfully plasmid-transfected cells. Due to the existence of endogenous TMEM43, it is difficult to separate the phenotype derived from TMEM43 mutant or endogenously. Making an endogenous model on TMEM43 mutant is highly recommended.

DAPI is often not mentioned in the figure legend where IF was described.

Figure 4:

1. VDAC is a transmembrane calcium channel protein expressed in the mitochondrial outer membrane. In Figure 4A, the red fluorescence of VDAC2 is from the nucleus. VDAC1 looks normal.
2. Why is HA localization very different from Figure 3? Isn’t TMEM localized in the nuclear membrane? In Figure 4A, HA is also expressed in the cytosol.

Therefore, the authors can’t make convincing results from Figure 4A.

Figure 4B, IgG control is needed.

Figure 4C, the description is not clear. Labeling what the samples are and what antibodies are used to precipitate is recommended.

For input samples, the TMEM43 density is too low to tell. Either increase the loading amount or use a more sensitive ECL.

All Western images need to be labeled the molecular size ladder; please refer to Western image publication standards. In the supplementary, please upload all raw images with appropriate labeling.

To conclude, the decreased interaction between VDAC and TMEM needs to be quantified. Another method is highly recommended to make the conclusion convincing. If authors can make IF results convincing, it also counts. Stain cells with mitochondria maker to see whether mitochondria co-localization with the nucleus decrease may be helpful as VDAC is expressed in mitochondria.

Figure 4A, I recommend having an individual channel in addition to a merged one; for example, the red fluorescence in the left bottom is too dim to tell whether there is some localization on the nuclear membrane or TMEM43.

There are limitations of IF and IP. IF is a 2D image, and proteins are inseparable between the cytosol and organelles if a generic protein lysis method is used. To make the data more convincing, nuclear protein-specific isolation is recommended to test whether the mutant group's intersection between VDAC and TMEM43 is decreased.

Figure 5:

A did not label what violet signal is.

Line 467, it is abrupt to mention TMEM43 function in mitochondria, it is not clearly described in the context.

B and C

Analysis should be done in the cells that have the plasmid transfected. Not all cells have the TMEM mutant plasmid.

Figure B needs a higher resolution to indicate that JC-1 is in the mitochondria to prove its efficacy.

Minor:

It is vague to say:

“IF staining of TurboID H9c2 cells revealed that  biotin and HA labeled TMEM43 were completely overlapping (Fig. S5), indicating  the precision of TurboID system.”

Are authors implying Turbo ID system consistency with Flag-tagged TMEM43 used before, since plasmids in both systems are HA-tagged?

The expression plasmid information in the flag tagged TMEM43 has not been revealed.
