# Supplementary material for: Proteomic screening of TMEM43 binding partners identifies VDAC leading to mitochondrial dysfunction
Source: PLoS One. 2025 Dec 18;20(12):e0339129. doi: 10.1371/journal.pone.0339129 (PMC12714281; doi:10.1371/journal.pone.0339129)
Supplement: Attachment — Submitted filename: Response to Reviewers 4.docx [file pone.0339129.s017.docx]

Please submit your revised manuscript by Sep 21 2025 11:59PM. If you will need more time than this to complete your revisions, please reply to this message or contact the journal office at plosone@plos.org. When you're ready to submit your revision, log on to https://www.editorialmanager.com/pone/ and select the 'Submissions Needing Revision' folder to locate your manuscript file.

Please include the following items when submitting your revised manuscript:

A rebuttal letter that responds to each point raised by the academic editor and reviewer(s). You should upload this letter as a separate file labeled 'Response to Reviewers'.

A marked-up copy of your manuscript that highlights changes made to the original version. You should upload this as a separate file labeled 'Revised Manuscript with Track Changes'.

An unmarked version of your revised paper without tracked changes. You should upload this as a separate file labeled 'Manuscript'.

Reviewer #2: There are still some concerns to be addressed:

- VDAC is a mitochondrial outer membrane protein. The search in genecard is not sufficient to prove that VDAC can both localize to mitochondria and nucleus. The authors should search in literature and cite the related findings. Moreover, only based on this, the authors cannot conclude that “indicates that VDAC may interacts with TMEM43 in the nucleus”, because some nuclear proteins are able to translocate to mitochondria.

Response：Sure, VDAC1 is a mitochondrial membrane protein that interact with other partners in mitochondria [1]. Hanna Galganska et al. predicted the communication of VDAC between mitochondria and nucleus [2]. Moreover, Sara de Mateo et al. discovered that VDAC1 and VDAC2 both located in human sperm nucleus through LC-MS/MS [3]. Our immunoblotting analysis showed expression of VDACs proteins in the nucleus (S8C Fig). TMEM43 locates to nucleus inner membrane [4]. Another possibility is that TMEM43 translocates to mitochondria and interacts with VDAC there. Therefore, we do not claim their interaction in the nucleus in the manuscript.

Reference：

[1] Shanmughapriya S, Rajan S, Hoffman NE, Higgins AM, Tomar D, Nemani N, Hines KJ, Smith DJ, Eguchi A, Vallem S, Shaikh F, Cheung M, Leonard NJ, Stolakis RS, Wolfers MP, Ibetti J, Chuprun JK, Jog NR, Houser SR, Koch WJ, Elrod JW, Madesh M. SPG7 Is an Essential and Conserved Component of the Mitochondrial Permeability Transition Pore. Mol Cell. 2015 Oct 1;60(1):47-62. doi: 10.1016/j.molcel.2015.08.009. Epub 2015 Sep 17. PMID: 26387735; PMCID: PMC4592475.
[2] Galganska H, Karachitos A, Wojtkowska M, Stobienia O, Budzinska M, Kmita H. Communication between mitochondria and nucleus: putative role for VDAC in reduction/oxidation mechanism. Biochim Biophys Acta. 2010 Jun-Jul;1797(6-7):1276-80. doi: 10.1016/j.bbabio.2010.02.004. Epub 2010 Feb 6. PMID: 20144586.
[3] de Mateo S, Castillo J, Estanyol JM, Ballescà JL, Oliva R. Proteomic characterization of the human sperm nucleus. Proteomics. 2011 Jul;11(13):2714-26. doi: 10.1002/pmic.201000799. Epub 2011 Jun 1. PMID: 21630459.
[4] Bengtsson L, Otto H. LUMA interacts with emerin and influences its distribution at the inner nuclear membrane. J Cell Sci. 2008 Feb 15;121(Pt 4):536-48. doi: 10.1242/jcs.019281. Epub 2008 Jan 29. PMID: 18230648.

In addition, the authors should perform immunoblotting analysis of VDAC in subcellular fraction.

Response: The immunoblotting analysis of VDACs in the nucleus (S8C Fig) showed that both VDACs expressed in the nucleus of H9c2 cells. And the IF staining of VDAC is overlapping with DAPI (Fig 4A).

-If the authors conclude VDAC interacts with TMEM43 in the nucleus, what is the molecular mechanism through which TMEM43 regulates mitochondrial dysfunction?

Response: The statement of “VDAC interacts with TMEM43 in the nucleus” is not included in the manuscript. Here is one hypothesis only for discussion with the reviewer, which is not even mentioned in the manuscript. Reduced binding of VDACs with TMEM43 mutant in the nucleus leads to higher level of VDACs in mitochondria, triggering excessive mPTP opening. Finally, this results in lower mitochondrial matrix Ca2+ level, higher mitochondrial ROS level and lost of mitochondrial membrane potential in mutation cells. Another possibility is not excluded. Wild type TMEM43 translocates from nucleus to mitochondria and interacts with VDACs. Mutation of TMEM43 reduces its binding to VDACs, leads to higher level of VDACs in mitochondria, and triggers excessive mPTP opening. Eventually, it results in the dysfunctions of mitochondria. Similar roles were identified in Hexokinase 1 (HK1) [5]. The decreased binding of VDAC1 to mutant HK1 led to lower level of mitochondrial matrix Ca2+.

Reference：

[5]Ceprian M, Juntas-Morales R, Campbell G, Walther-Louvier U, Rivier F, Camu W, Esselin F, Echaniz-Laguna A, Stojkovic T, Bouhour F, Latour P, Tricaud N. The Hexokinase 1 5'-UTR Mutation in Charcot-Marie-Tooth 4G Disease Alters Hexokinase 1 Binding to Voltage-Dependent Anion Channel-1 and Leads to Dysfunctional Mitochondrial Calcium Buffering. Int J Mol Sci. 2024 Apr 15;25(8):4364. doi: 10.3390/ijms25084364. PMID: 38673950; PMCID: PMC11050395.

-In the original Figure 4C, the input of VDAC1 in TMEM43 group and all the TMEM43 bands are weak. The authors only changed the blots of input but not the corresponding blots of IP, which is not convincing. The authors should replace all the blots in the original Figure 4C with new experimental data.

Response: All the blots in the original Figure 4C are replaced with new experimental data in Fig 4D.

-The authors should describe the mitochondrial Ca2+ level measurement and explain why there is no difference between WT and TMEM43 mutant.

Response: The mitochondrial matrix Ca2+ level was detected through Fluo-4 AM fluorescent probe, please refer to “Detection of Ca2+ level” in method section at page15. The results are renewed in Fig 5C. The mitochondrial matrix Ca2+ level decreased in H9c2 cells of TMEM43 mutant.

-The role of mitochondrial permeability transition pore in the discussion is not sufficient.

Response: The roles of mPTP are demonstrated at page 23 with 6 references.
