# Supplementary material for: Proteomic screening of TMEM43 binding partners identifies VDAC leading to mitochondrial dysfunction
Source: PLoS One. 2025 Dec 18;20(12):e0339129. doi: 10.1371/journal.pone.0339129 (PMC12714281; doi:10.1371/journal.pone.0339129)
Supplement: Attachment — Submitted filename: Response to Reviewer#2 d.docx [file pone.0339129.s018.docx]

Reviewer #2: The authors have addressed most of my concerns, but there are still some minor revisions needed:

1)Mitochondria are closely associated with nucleus. The purity of nucleus in S8C Fig need to be checked using mitochondrial markers like TOM20 and ANT etc.

Response:

We thank the reviewer for their valuable comments on this issue. After optimizing and repeating our experiments (≥3 times), we found that it remains technically challenging to completely remove TOM20-positive mitochondrial proteins from nuclear lysates.

Interestingly, a German group recently reported that [1] “Drosophila Tmem43 is localized at the ER/SR (endoplasmic reticulum/sarcoplasmic reticulum) membrane and interacts with the outer mitochondrial membrane protein Porin/VDAC. This interaction is lost in a Tmem43 p.S333L mutant that resembles the human p.S358L mutation. In addition, Tmem43 p.S333L caused a breakdown in mitochondrial membrane potential and increased cellular reactive oxygen species.”

One possible explanation for the presence of TOM20 in wild-type cells but not in mutant cells is that ER/SR–mitochondria complexes are present in the nuclear lysates of wild-type TMEM43 cells, whereas the TMEM43 mutation disrupts ER/SR–mitochondria association, leading to the loss of mitochondria from nuclear lysates.

Based on these findings, we propose the following molecular mechanism: under physiological conditions, TMEM43 located in the ER/SR interacts with VDACs on the mitochondrial membrane. The TMEM43 p.S358L mutation impairs this binding capacity, thereby disrupting ER/SR–mitochondria tethering. This, in turn, triggers mitochondrial dysfunction, manifested as mitochondrial fragmentation, loss of membrane potential, reduced Ca²⁺ levels, and elevated ROS.

2)“IF staining of VDAC is overlapping with DAPI” is not sufficient to conclude that VDAC can be localized in nucleus but may be just associated or interacted with nucleus.

Response: It is possible that VDAC may be just associated or interacted with nucleus.

3)The statement of “VDAC interacts with TMEM43 in the nucleus”is not included in the manuscript. This hypothesis should be discussed in the discussion section.

Response: This hypothesis is updated in the discussion section of updated manuscript. That is “It has been reported that Tmem43 interacts with VDACs at the endoplasmic/sarcoplasmic reticulum-mitochondrial (ER/SR-mitochondrial) contact sites, and this interaction is essential for normal mitochondrial function.”

4)Mutation of TMEM43 reduces its binding to VDACs, leads to higher level of VDACs in mitochondria, and triggers excessive mPTP opening. Is there any evidence that high VDAC can trigger PTP opening?

Response: Indeed, several indirect lines of evidence support a role for elevated VDAC levels in triggering PTP opening. The relationship is evidenced by both gain-of-function and loss-of-function studies:

· On one hand, VDAC1 overexpression induces cell death, and higher VDAC1 levels correlate with increased apoptosis in pathological models [2, 3].

· On the other hand, down-regulating or inhibiting VDAC1 produces protective effects, such as suppressed cell growth and reduced injury during ischemia-reperfusion [2, 4].

· Mechanistically, VDAC inhibition with antibodies prevents pivotal mitochondrial apoptotic events, including cytochrome c release and the collapse of the membrane potential [5].

Based on this evidence, it has been proposed that VDAC is a key contributor to PTP opening, which subsequently leads to mitochondrial damage and cell death [6]. This body of work indirectly supports our observation that increased VDAC levels could facilitate mPTP opening.

References

[1] Jürgens K, Menzel L, Klinke N, Schäper L, Breitsprecher L, Holtmannspötter M, Psathaki OE, Walter S, Ratnavadivel S, Malmendal A, Meyer H, Milting H, Paululat A. The ARVC-5-associated protein TMEM43 controls mitochondrial energy metabolism by stabilising ER-mitochondrial contact sites. Cell Mol Life Sci. 2025 Nov 14;82(1):400. doi: 10.1007/s00018-025-05942-z. PMID: 41236655; PMCID: PMC12618784.

[2] Abu-Hamad, S.; Sivan, S.; Shoshan-Barmatz, V. The expression level of the voltage-dependent anion channel controls life and death of the cell. Proc. Natl. Acad. Sci. USA 2006, 103, 5787–5792.

[3] Sasaki, K.; Donthamsetty, R.; Heldak, M.; Cho, Y.-E.; Scott, B.T.; Makino, A. VDAC: Old protein with new roles in diabetes. Am. J. Physiol. Physiol. 2012, 303, C1055–C1060.

[4] Feng, Y.; Madungwe, N.B.; Imam Aliagan, A.D.; Tombo, N.; Bopassa, J.-C. Liproxstatin-1 protects the mouse myocardium against ischemia/reperfusion injury by decreasing VDAC1 levels and restoring GPX4 levels. Biochem. Biophys. Res. Commun. 2019, 520.

[5] Shimizu S, Matsuoka Y, Shinohara Y, Yoneda Y, Tsujimoto Y. Essential role of voltage-dependent anion channel in various forms of apoptosis in mammalian cells. J Cell Biol. 2001 Jan 22;152(2):237-50. doi: 10.1083/jcb.152.2.237. PMID: 11266442; PMCID: PMC2199613.

[6] Parmar MY, Shah PA, Gao J, Gandhi TR. Hepatoprotection through regulation of voltage dependent anion channel expression by Amomum subulatum Roxb seeds extract. Indian J Pharmacol. 2011 Nov;43(6):671-5. doi: 10.4103/0253-7613.89824. PMID: 22144772; PMCID: PMC3229783.
